# Research on the Cold Rolling Process, Microstructure and Properties of 305 Austenitic Stainless Steel Thin Strips

**DOI:** 10.3390/ma17061250

**Published:** 2024-03-08

**Authors:** Huanhuan Wang, Lifang Pan, Yong Chen, Zhihui Cai, Yongshun Zhao, Guangming Liu

**Affiliations:** 1 School of Materials Science and Engineering, Taiyuan University of Science and Technology, Taiyuan 030024, China; wanghuanhuan0728@163.com (H.W.); 2013017@tyust.edu.cn (L.P.); ychen@tyust.edu.cn (Y.C.); 2 School of Mechanical Engineering, Taiyuan University of Science and Technology, Taiyuan 030024, China; 2020050@tyust.edu.cn; 3 R&D Center, Shanxi Taigang Stainless Steel Precision Strip Co., Ltd., Taiyuan 030003, China; zt189994@163.com

**Keywords:** stainless steel, cold rolling, martensitic phase transformation, annealing treatment, magnetic permeability

## Abstract

Austenitic stainless steel has high toughness and plasticity; however, it tends to exhibit low yield strength, which severely limits the widespread application of this steel. It can be strengthened by cold working; however, this will cause many defects in the structure. Therefore, annealing treatment must be carried out before use. In this paper, the effects of annealing treatment at different temperatures and times on the microstructure and mechanical properties of cold-rolled 305 stainless steel sheet were studied and the theoretical mechanism was further analyzed to provide better theoretical guidance for production and application. It was found that the microstructure grains obtained by annealing at 850 °C for 30 s were finer and more uniform, and the mechanical properties were also the best, which met the requirements of strong plasticity. Therefore, the rolling and annealing experiments could be carried out again under this annealing condition, and the requirements of the finished product could be finally obtained. At this time, the thickness of the plate was about 0.15 mm, the yield strength was 1238 MPa, and the permeability was below 1.02, which met the production requirements of the metal mask plate.

## 1. Introduction

With the rapid development of social economy in recent years, the demands for austenitic stainless steels have shown an explosive growth trend worldwide. Meanwhile, with their own unique advantages, such as high plasticity and toughness, strong corrosion resistance and good cold-forming performance at room temperature, austenitic stainless steels have been widely used in various fields [1], such as medical equipment, aerospace, daily life, and the petrochemical industry. Particularly, some austenitic stainless steels, such as 305 austenitic stainless steel, have been adopted in the production of high-end electronic products. Therefore, these kinds of steels have attracted much attention worldwide, especially in China where some high-end electronic products need to be imported, and among which the 5G communications metal mask templates are all dependent on imports.

However, what needs to be pointed out is that due to the low yield strength, the applications of 305 austenitic stainless steel in the production of high-end electronic products are extremely limited. As a consequence, the improvement of yield strength is the first concern in the research of 305 austenitic stainless steel for high-end electronic product applications.

It is well known that cold rolling (during which severe plastic deformation usually occur) is a feasible and effective method to improve the yield strength of metals and alloys. Therefore, a lot of attention has been paid on the research of the cold rolling of austenitic stainless steels by the scholars all over the world [2,3]. The results indicate that deformation twins and slip lines could be formed during cold rolling, and micro-twins could impede dislocation movement, resulting in the increase in yield strength. The development of ultrafine grain structure in metastable austenitic stainless steel grades is of utmost importance as it provides the potential for utilizing high-strength austenitic stainless steels in structural applications. Therefore, the origin and morphology of martensite in cold-rolled austenitic stainless steels have also been extensively studied. Olson et al. and Tamura [4] discussed a phenomenological model that could be used to explain the formation of strain-induced martensite caused by cold rolling; also the results of the study showed that the martensitic morphology in low-carbon steel, special steel, and austenitic stainless steel after cold rolling deformation mainly consists of dislocated forests, dislocated walls and tangles, large deformed lath martensite, and dislocated cytoplasmic martensite. Jana, Weyman, Coleman and West discussed the mechanism of phase transformation leading to changes in the magnetic properties of alloys [5]. Guy et al. investigated the phase transformation mechanism from a crystallographic point of view, while Breedis and Krauss reported the formation of twins and laminar dislocations due to phase transformation [6]. Recent studies have demonstrated that the formation of ultrafine austenite grains in austenitic stainless steels can be achieved by cold roll annealing, which improves the strength of the material, while other studies on substable austenite have demonstrated the feasibility of this research method [7]. Lee et al. [8] investigated pure nickel by using a cold rolling and annealing process, and found that annealing experiments carried out on it at the appropriate temperatures were effective in refining the grains to improve the strength. Rangaraju et al. [9] similarly refined the grains by annealing experiments. There are many studies on grain refinement of austenitic stainless steel using cold rolled annealing process to improve the strength of the material, but very few studies have been carried out to regulate both strength and permeability during the annealing process.

Based on the results of the above studies, it can be seen that the strength of austenitic stainless steel can be effectively improved by cold rolling, but the resulting martensitic phase will have an impact on the magnetic permeability. Although there are many studies on the cold rolling and annealing process of austenitic stainless steel, there are few studies on the regulation of strength and magnetic permeability at the same time, so a more in-depth study is necessary. Also, there is no or very little research on cold roll annealing of 305 stainless steel, so there is a need for research on 305 stainless steel. In addition, due to the special chemical composition of 305 austenitic stainless steel, deformation-induced martensitic transformation will occur during cold processing, resulting in changes in the magnetic permeability and mechanical properties of this material. If the magnetic permeability exceeds a certain limit, the transmission of signals will be shielded, thus it is also essential to regulate the magnetic properties of 305 stainless steel material during cold deformation process.

In this paper, the cold rolling and annealing experiments of 305 austenitic stainless steel were carried out to study the microstructure evolution, mechanical properties and magnetic properties of this steel under different conditions, so as to provide theoretical basis and technical support for the development of new stainless steel.

## 2. Materials and Methods

### 2.1. Experimental Materials

The experimental materials are selected as 305 austenitic stainless steel thin strips (Provided by Shanxi Taigang Stainless Steel Precisoin Strip Co., Ltd., Taiyuan, China); the initial thickness of the materials is 1 mm, and their chemical composition is shown in Table 1.

### 2.2. Process Routes

**Figure 1:**
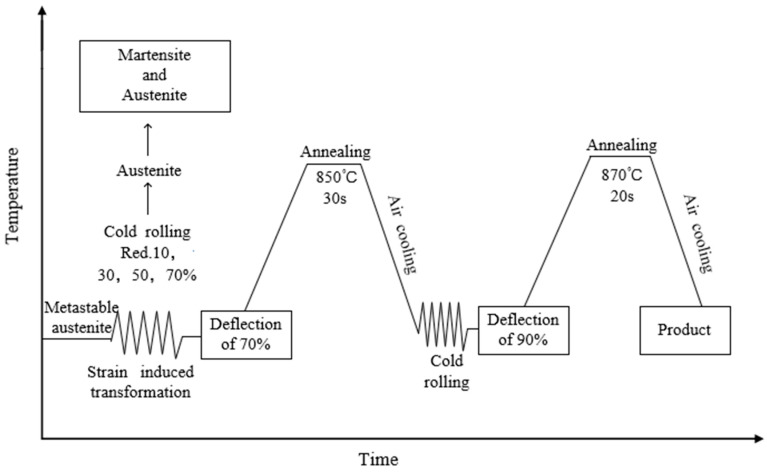
Process flow diagram.

The multi-pass cold rolling experiments were carried out on a six-high reversible rolling mill. The linear speed of the roll during the rolling process was 0.1 m/s, and the cumulative deformation was 70%. At this time, due to the serious strengthening, the subsequent rolling experiment can not be carried out. Therefore, the annealing experiment was carried out on the experimental steel with a deformation of 70%. The experimental steel was annealed at 800, 850 and 900 °C for 10, 30, 60 s, respectively, and then was air-cooled to room temperature. After annealing at 850 °C for 30 s, rolling was carried out again; after rolling, the thickness and strength of the strip reach the requirements but the permeability exceeds the standard, therefore, an annealing experiment was carried out to control the permeability. At this time, the appropriate annealing process is 870 °C for 20 s. The process diagram is shown in Figure 1.

### 2.3. Microstructure Observations, Tensile Testing and Hardness

The specific characterization and testing methods for the microstructure and mechanical properties of the experimental steels are as follows: the metallographic specimens were sampled along the rolling direction, mechanically ground and polished, and then corroded with aqua regia solution for 30 s. Afterwards, they were used for optical microscope (OM, DMM-2000C, Made in G-TECH, Zhuhai, China) and scanning electron microscope (SEM, a JEOL JEM6700, Made in Akishima, Japan) observations. At the same time, the experimental steel was prepared into a wafer with a thickness of 30 μm by wire cutting and the subsequent mechanical grinding. After punching and electrolytic double spraying, the wafer was prepared into a standard transmission sample with a size of φ = 3 mm, which was used for the observation of dislocation configuration and the statistics of dislocation density. The tensile specimens were designed according to the national standard GB/T228.1-2021 [10] and subsequently cut. The equipment used in the tensile tests were DNS200 (Made in Kimlida Electronic Technology Co., Ltd., Guangdong, China) universal testing machine, which were carried out at room temperature with a tensile speed of 1 mm/min. The tensile tests were carried out three times for each data point, and the average values were taken to ensure the accuracy of the experimental results.

The specimens were sequentially sanded smooth and flat on 800 mesh to 1500 mesh sandpaper, measured on the Vickers microhardness tester (HV-1000Z, Made in Jinan Kason Testing Equipment Co., Ltd., Jinan, China) with a load of 300 N and a holding pressure of 15 s, and the lengths of the two diagonals of the rhombus-shaped spots were measured so as to calculate the Vickers hardness, and each specimen was randomly taken at different locations under the same conditions for about 3 points, and the average value was taken as the final result.

The plates were further analyzed by X-ray diffraction to determine the phase composition of the plates after rolling. They were ground using 1500-mesh sandpaper. It was then polished and cleaned with alcohol after polishing to remove contaminants. The sample was then fixed to the work table to better focus the X-ray beam. The equipment used for the X-ray diffraction analyses was a Japanese Ultima IV series with a scanning rate of 2°/min, a step size of 0.02°, and a scanning angle in the range of 2θ = 40 to 120°. The obtained peaks 2θ were plotted as a function of the corresponding function. The peaks were indexed and the peak intensity obtained using MDI Jade 6.5 software.

The procedure for the permeability test was to place a strip of alloy of approximately 60 mm in length into a solenoid in order to ensure that the demagnetization factor remained constant during the test. The strip was weighed and recorded before the permeability test. Since the length of the strip is much greater than the width and thickness of the strip, it can be assumed that the length has little effect on the coercivity and permeability.

## 3. Analysis of Rolling Test Results

### 3.1. Organizational Characterization

#### 3.1.1. Microstructure Characterization

**Figure 2:**
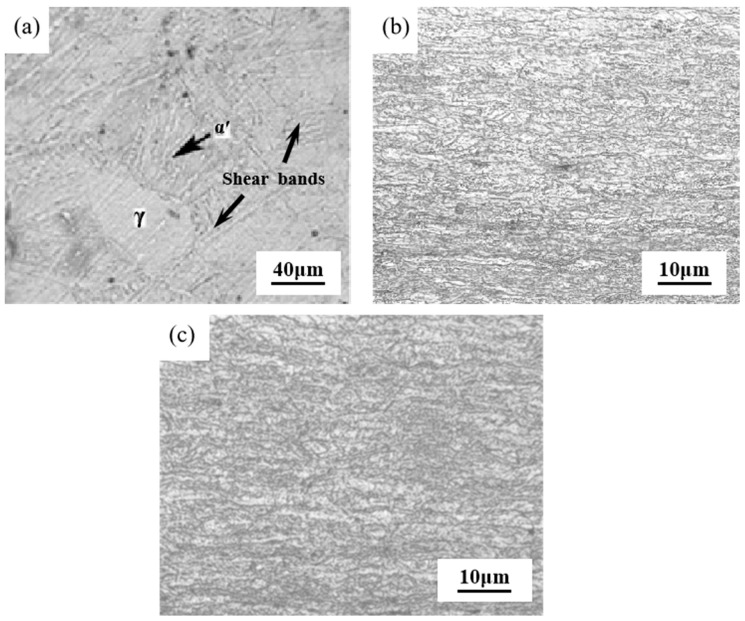
Metallographic organization of austenitic stainless steels under different cold rolling deformations. (**a**) deformation of 10%; (**b**) deformation of 50%; (**c**) deformation of 90%.

In the process of cold rolling deformation, the degree of grain deformation increases with the increase of deformation [11]. From the overall deformation trend of the grains, as the deformation increases step by step, most of the grains have undergone severe plastic deformation, and the grains have been divided into many fine grains. The equiaxed austenite with clear grain boundaries [12] has basically disappeared, and the complete grains have not been seen. The grains have a more serious slip phenomenon, and the slip direction is along the rolling direction, and finally the grains become long strips (Figure 2c). With the increase of cold rolling deformation, the degree of grain elongation is more and more intense, and the finer the grain is, the change of microstructure is caused, so that the performance of stainless steel also changes accordingly.

In terms of microstructure changes, in austenitic stainless steel, the austenitic phase γ (fcc), which is a high-temperature phase of iron-carbon alloys, is usually an unstable phase [13]. At the very beginning of the deformation stage, the shear bands composed of stacking faults and α’-martensite (bcc) are formed in austenitic stainless steel, and the interface of the shear bands provide the nucleation site for the formation of α’-martensite (bcc).With the increase of deformation, the austenitic phase gradually transforms into α’-martensite. Many shear bands are found in the austenite phase when the cold rolling reduction is 10% (Figure 2a). Strain-induced martensite is formed at the shear band when the cold rolling reduction is increased to 50% [14], and when the cold rolling reduction continues to increase to 90%, the untransformed austenite is further elongated along the direction of rolling to a banded organization (Figure 2c). And with the gradual increase in the cold rolling reduction, the austenite is further segmented, which leads to a further increase in the amount of martensite as well.

The transformation of austenite to martensite is due to the fact that the shear band provides a potential nucleation [15] site for α’-martensite, and α’-martensite starts to nucleate and grow at the shear band and gradually takes the place of the shear band. At the same time, with the increase of deformation, the number of shear bands are gradually increasing, resulting in an increase in the volume fraction of martensite. The untransformed austenite is gradually divided into fine-grained structure by martensite. When the amount of deformation is increased to a certain degree, the grains are deformed to form fibrous structure. The deformation-induced martensite is severely deformed or even broken, resulting in the formation of dislocation cellular martensite, compared with other martensites, this martensite dislocation density is high.

#### 3.1.2. XRD Analysis

**Figure 3:**
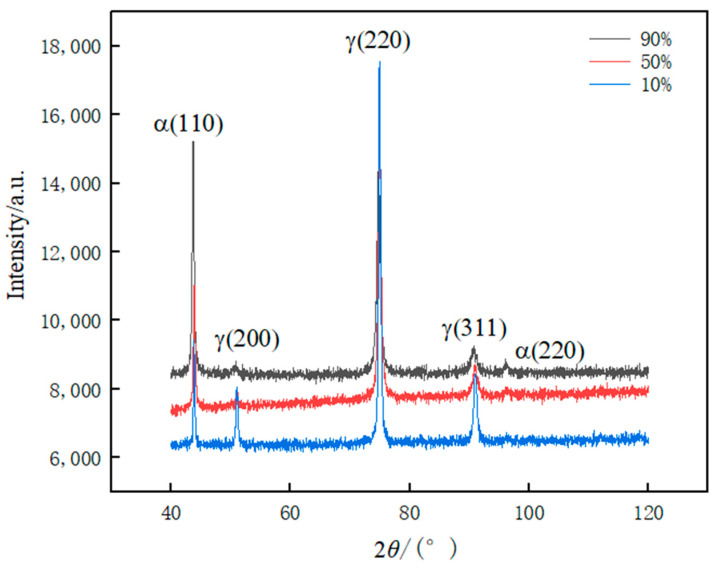
X-ray diffraction pattern of plates with different cold-rolled deformation.

Figure 3 shows the X-ray diffraction analysis under different deformation after cold rolling. It is obvious from the figure that in addition to the austenite peaks, (110) and (220) peaks associated with the α′-martensite phase also appear in the diffraction spectrum of the cold-deformed sample. The diffraction peaks of the γ-phase and the α′-martensite phase can be seen in each of the diffraction patterns [16], which indicates that the phase transformation has occurred in the process of the cold-rolling deformation at each deformation amount. From the figure, it can also be observed that when the amount of deformation is small, the α′-martensite peak will not be detected, this is because at room temperature and at low levels of deformation, the martensite content is very low, it is difficult to analyze it quantitatively by X-ray diffraction [17]; as the amount of deformation is increased, higher deformations begin to induce the austenite-to-martensite transformation. Figure 2a shows that the formation of α′-martensite is the main manifestation of deformation-induced phase transformation. It can also be observed that the diffraction peak intensity of the γ-phase gradually becomes weaker and that the α′-martensite phase gradually increases due to the increase in rolling deformation, indicating that the content of the γ-phase decreases with the increase of rolling deformation, while the content of the α′-martensite phase increases with the increase of rolling deformation. In addition, with the increase of rolling deformation, the diffraction peak width of γ-phase gradually increases. The main reason is that with the increase of rolling deformation, the residual stress and grain refinement degree inside the rolled plate gradually increase, and the number of dislocations that are piled up or delivered also gradually increases [18].

To calculate the corresponding martensite content based on X-ray diffraction analysis, the following specific calculation formula is used:

(1) $$V_{\gamma }=\frac{1.4I_{\gamma }}{I_{\alpha }+1.4I_{\gamma }}$$

where, $V_{\gamma }$ is the volume fraction of austenite; $I_{\gamma }$ is the integral intensity of the diffraction peak of austenite at the crystal plane; $I_{\alpha }$ is the integral intensity of the ferrite diffraction peak at the crystal plane. The volume fraction of martensite can be obtained according to that of austenite. Table 2 shows the content of austenite (fcc) and α′ -martensite (bcc) phase obtained by analysis and calculation. From Table 2, it is clear that a higher content of α′-martensite appears with the increase of cold deformation.

#### 3.1.3. TEM Analysis

**Figure 4:**
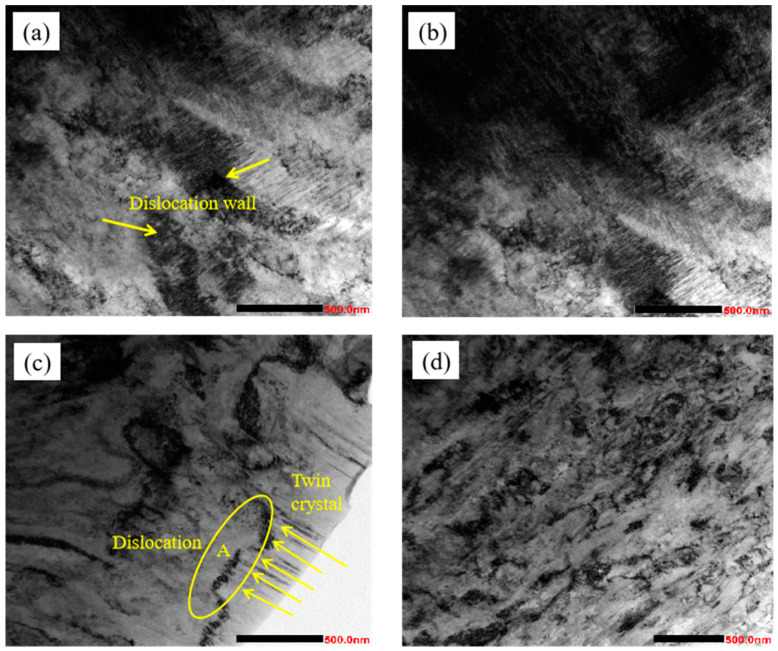
TEM organization of austenitic stainless steels under different cold rolling deformations. (**a**) deformation of 30%; (**b**) deformation of 50%; (**c**)deformation of 70%; (**d**) deformation of 90%.

With the increase of cold rolling deformation, the degree of plastic deformation of grain becomes more and more intense. Figure 4 shows the microstructure morphology of the experimental steel under different cold rolling deformation. It can be seen from the figure that after cold rolling deformation, a large number of mutually entangled dislocations [19] are generated inside the sample, and the degree of entanglement gradually increases. When the deformation increases to a certain extent, deformation twins will appear. When the deformation is 30%, a large number of dislocations are generated inside the sample, and the dislocations are entangled with each other, gradually forming a dislocation wall, as pointed out by the arrows in Figure 4a; as the deformation increases to 50%, it can be seen that the dislocation density inside the sample increases sharply, and the degree of dislocation entanglement becomes more and more intense, as is shown in Figure 4b; when the deformation continues to increase to 70%, the degree of dislocation entanglement is further aggravated, and a large number of deformation twins appear, as indicated by the arrows in Figure 4c, and the dislocations and twins are intersected with each other, as is shown in region A of Figure 4c. After the deformation increases to 90%, the dislocations and twins intersect with each other, so that the grains of the experimental steel have been completely fragmented to the nanometer scale, as is shown in Figure 4d.

The formation of different microstructure morphologies is closely related to the different cumulative strain under different deformation of cold rolling. When the deformation is small, there is only the proliferation and accumulation of dislocations and the interactions between dislocations, and when the deformation is further increased, the interactions between dislocations will be obviously aggravated due to severe plastic deformation, which will make the slip movement of dislocations difficult to proceed, and then the interactions between dislocations will promote grain fragmentation, so that the dislocations density will gradually decrease. Severe plastic deformation will also lead to the formation of a large number of lamellar stacking faults, and the thicknesses of these stacking faults are close to those of submicroscopic twins, which are the precursor of twins [20]. When the deformation reaches a certain degree, stacking faults will be transformed into lamellar twins. Through the observation of the cold rolling process of stainless steel, it is also found that in the process of cold rolling deformation of high nitrogen austenitic stainless steel, when the deformation amount was small, the microstructure was mainly dislocation slip and dislocation proliferation and accumulation, and when the deformation amount increased, the microstructure was mainly deformation twins. In fact, in any stage of rolling, dislocation slip, dislocation multiplication, dislocation interaction and deformation twins exist at the same time, but the proportion of different deformation stages is different, these combined effects make the austenite grains refine continuously.

### 3.2. Characterization of Mechanical Properties

**Figure 5:**
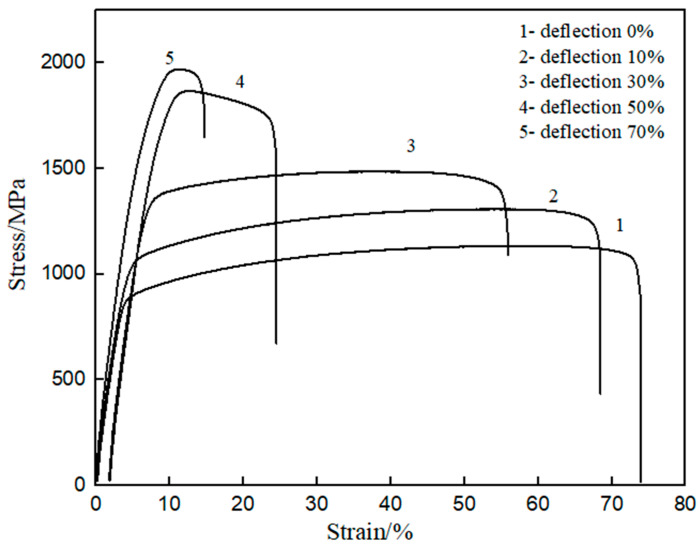
Engineering stress–strain curves of the plates after cold rolling to different amounts of deformation.

**Figure 6:**
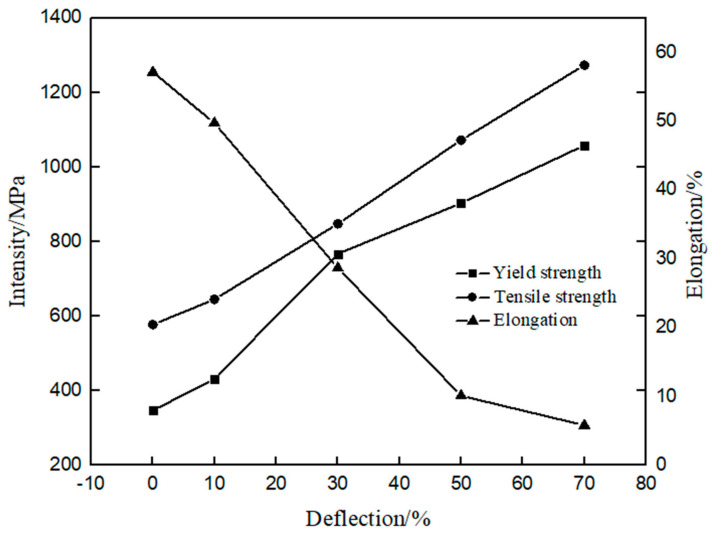
Tensile test data of the plates after cold rolling to different amounts of deformation.

The engineering stress–strain curves of the plates at different deformations after cold rolling are shown in Figure 5. The values of the material-related mechanical properties obtained from the engineering stress–strain curves are shown in Figure 6. It can be seen that with the increase of rolling deformation, the yield strength and tensile strength of the plates increases; however, the total elongation shows an opposite trend. In particular, when the deformation is 70%, the yield strength reaches 1058 MPa, the tensile strength reaches 1273 MPa, the yield-strength ratio is about 0.83, and the total elongation drops to 5.82%. The results shows that the tensile strength increases with the increase of deformation, which in turn verified the correctness of the conclusions.

The microstructure of a material determines its mechanical properties to a large extent [21]. The proportions of austenite and martensite phases, grain size, and microstructure of the samples with different deformations have a great influence on their mechanical properties. On the one hand, dislocation diffusion occurs during cold rolling, which generates high stress energy in the material, increases its free energy, and promotes the transformation of austenite to α’-martensite. The crystal structure type of austenite is face-centered cubic, and the crystal structure type of α’-martensite is body-centered cubic. The latter has less slip direction and greater lattice resistance, that is to say, the α’-martensite phase is more difficult to slip. In the macro sense, the increase of α’-martensite can improve the strength and hardness. Therefore, the martensite phase can increase the strength and hardness of the sample, while the austenite phase favors the elongation of the specimen, and it also reveals that the phase ratio has a great influence on the mechanical properties of the sample. On the other hand, with the increase of deformation, the size of untransformed austenite is divided and refined, the density of grain boundaries is high, and the dislocation motion is greatly restricted. According to the Hall-Petch relationship [22], with the decrease of grain size and the increase of grain boundary density, the yield strength of the sample can be improved. This is because as the decrease in grain size and the increase in grain boundary density, the high-density grain boundaries effectively impede the movement of dislocations, and the ability of the grain boundaries to accommodate dislocations decreases, leading to an increase in yield strength.

In the macro sense, the increase of a’-martensite can improve the strength and hardness. Therefore, the martensite phase can increase the strength and hardness of the sample, while the austite phase favors the elongation of the specimen, and it also reveals that the phase ratio has a great influence on the mechanical properties of the sample. Therefore, there are two main aspects to improve the yield strength of 305 austenitic stainless steel during cold rolling: the first is deformation-induced martensitic phase transformation, the second is martensitic refinement of untransformed austenite grains, and the increase of hardness is the result of deformation-induced martensitic phase transformation and the increase of dislocation density.

**Figure 7:**
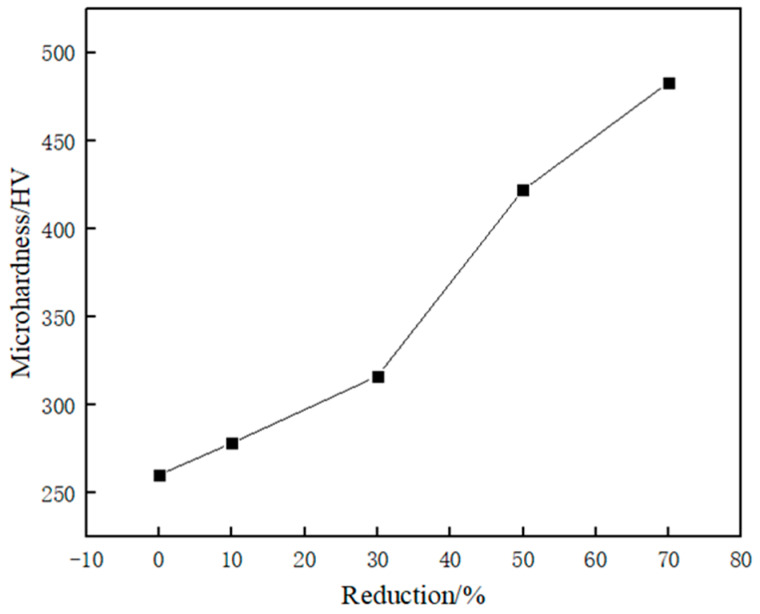
Microhardness at different deformations.

Figure 7 shows the hardness values of austenitic stainless steel after cold rolling deformation at different deformation amounts. From the figure, it can be seen that with the increase of cold rolling deformation, the hardness value of austenitic stainless steel is also gradually increasing. The hardness of the unrolled austenitic stainless steel specimen is about 260 HV; when the deformation is increased to 70%, the hardness value is about 480 HV, compared with the original unrolled austenitic stainless steel specimen, the increase is as high as 84%. It can be seen that with the increase of deformation, the hardness value of the austenitic stainless steel specimen is an increasing trend. The reason for the change in the hardness value is mainly in the cold rolling process of intense plastic deformation will make the organization form a large number of dislocations. dislocations entangled with each other, the formation of dislocation cells, with dislocation entanglement and dislocation cells of further slippage gradually dislocations appeared to be plugged, dislocations are difficult to carry out the movement, and at the same time, as the deformation continues to increase, the dislocations between the interactions increased significantly, so that the degree of hardening intensified, resulting in an increase in hardness. resulting in an increase in the hardness value.

### 3.3. Magnetic Characterization

**Figure 8:**
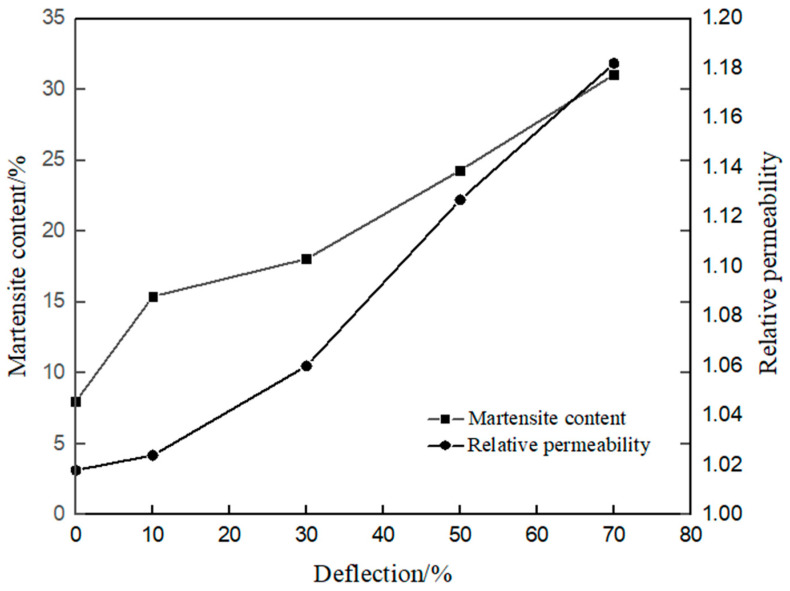
Permeability test data of the plates after cold rolling to different amounts of deformation.

Magnetic permeability detector is used to measure the change in magnetic permeability of the experimental steel under different cold rolling reduction rate; X-ray diffraction patterns are used to calculate the content of deformed martensite; according to the content of martensite after deformation, the corresponding curves can be drawn. Figure 8 shows that when the volume fraction of martensite is 31%, the relative permeability is 1.18.

After cold rolling deformation, the martensite phase will form in the experimental steel. Since the martensite phase has strong magnetism, the magnetic properties of the experimental steel will change. It can be seen from Figure 8 that the deformed martensite will appear during the cold rolling process. At this time, the martensitic transformation is a non-diffusion way, and the lattice transformation is shear. In general, the nucleation sites of deformed martensite will appear in shear bands, grain boundaries and other places, which is mainly related to various lattice defects in the crystal structure. With the increase of deformation, the content of martensite will increase, which leads to the increase of permeability.

## 4. Analysis of the Results of Annealing Experiments

When the deformation reaches 70%, due to serious work hardening, it is impossible to carry out rolling experiments with greater deformation. Combined with the analysis of rolling experimental results, it can be seen that the main reasons for the continuous increase of the yield strength are twin strengthening, dislocation strengthening and fine-grain strengthening. Combining the factors of strengthening, reasonable annealing experiments can be carried out to make it undergo reverse transformation of phase transformation, so that further rolling experiments can be carried out after softening, so that it can meet the production requirements.

### 4.1. Characterization of Annealed Microstructure

**Figure 9:**
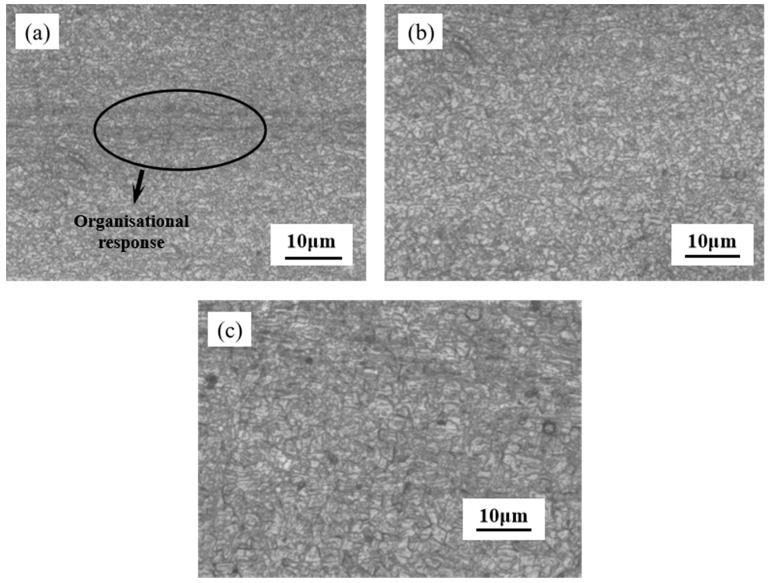
Microstructure of plates at different annealing temperatures. (**a**) annealed at 800 °C for 30 s; (**b**) annealing at 850 °C for 30 s; (**c**) annealing at 900 °C for 30 s.

The microstructure of the experimental steel with cold rolling deformation of 90% after annealing at different temperatures for 30 s are shown in Figure 9. It can be seen from the figure that when the annealing temperature is below 850 °C, the deformed structure only recovers [23]. When the annealing temperature is higher than 850 °C, the deformed structure begins to recrystallize, and with the increase of annealing temperature, the degree of recrystallization increases.

**Figure 10:**
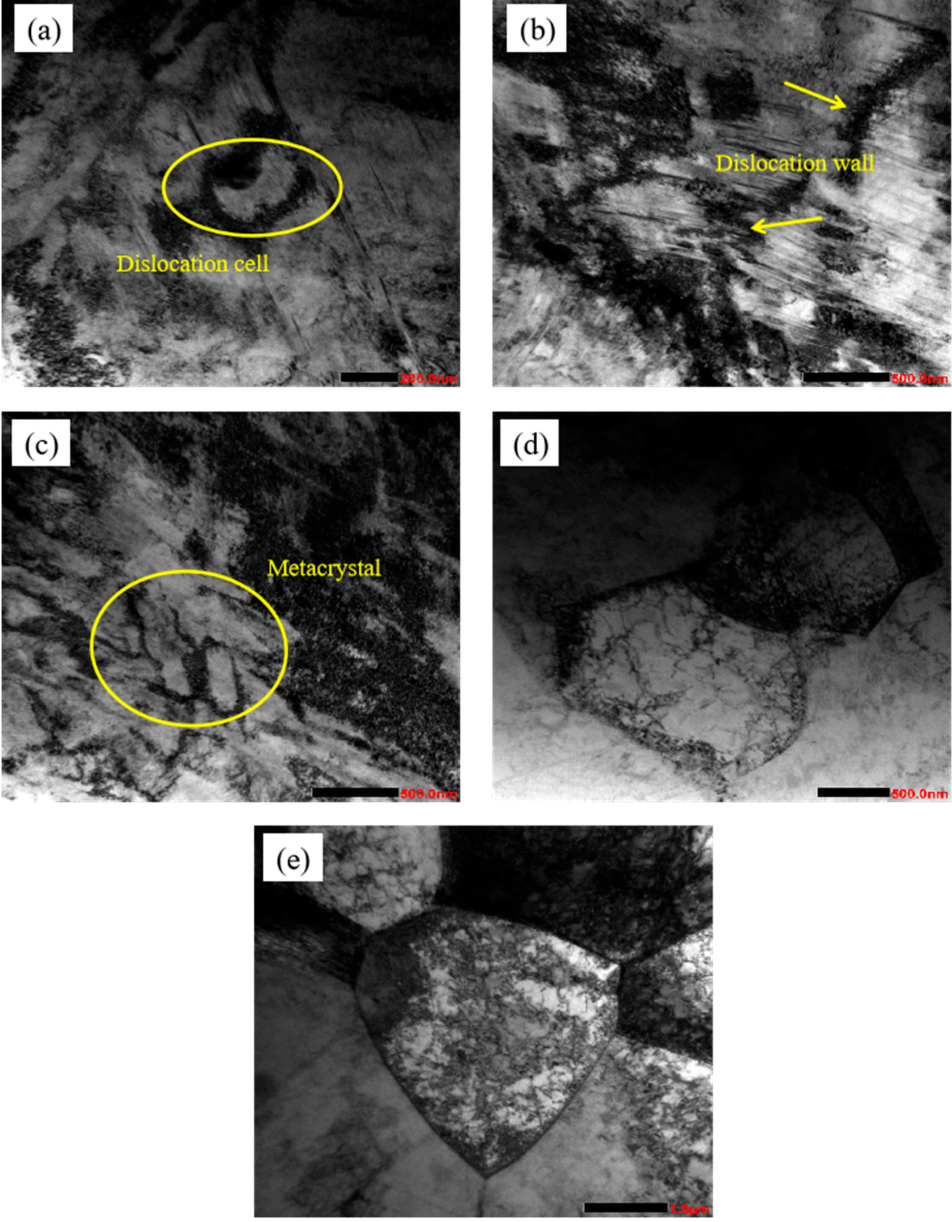
TEM organization of austenitic stainless steels at different annealing temperatures. (**a**) annealed at 750 °C for 30 s; (**b**) annealing at 800 °C for 30 s; (**c**) annealing at 850°C for 30 s; (**d**) annealed at 900 °C for 30 s; (**e**) annealing at 950 °C for 30 s.

When the annealing temperature is 750 °C, the dislocations move and recombine with each other to form dislocation cells, and the cell walls are composed of high-density dislocations, as is shown in Figure 10a. When the annealing temperature is 800 °C, the dislocation cell walls gradually disappear through the reaction between dislocations, the dislocation density gradually decreases, and finally the dislocation walls are formed, indicating that the deformed structure begins to enter the recovery stage at this time, as is shown in Figure 10b. When the annealing temperature increases to 850 °C, a large number of dislocations continue to move and interact with each other, and then the dislocation walls gradually disappear, forming polygonal subgrain structures with clear interface subgrain boundaries, as is shown in Figure 10c. When the annealing temperature is 900 °C, some grains have completely recrystallized and clear grain boundary structures are formed, as is shown in Figure 10d. When the annealing temperature continues to rise to 950 °C, the grain size increases significantly, as is shown in Figure 10e. As the annealing temperature continues to increase, the degree of grain recrystallization increases, and an equiaxed austenite structure with uniform structure and clear grain boundaries will be formed. According to the dislocation theory, the subgrain boundary is a grain boundary formed by dislocations. At a certain temperature, the subgrain boundary will slip and interact with the movement of dislocations and disappear. Therefore, the subgrain boundary continues to move and migrate, and gradually evolves into a large-angle interface, leading to the formation of recrystallized nucleus. With the increase of annealing temperature, the recrystallized grains continue to grow, and finally the equiaxed [24] austenite structure with clear grain boundaries is formed.

As the increase in annealing time, the content of martensitic phase decreases, while the grain size of which gradually increases. Both annealing temperature and time have an effect on the increase of grain size, and the annealing temperature has a more pronounced effect on grain growth.

The relationship between grain size and annealing time under isothermal annealing conditions can be expressed by the following Beck’s formula [25] for grain growth kinetics:

D − D_0_= kt^n^ (2)

where D is the grain size after annealing, D_0_ is the grain size before annealing, k is the rate parameter of grain growth, n is the kinetic index of grain growth; t is the annealing time. It is obvious from the formula that the grain size increases with the extension of the annealing time.

### 4.2. Mechanism of Martensite Austenite Reversal

It is generally accepted that the main purpose of reverse phase transformation annealing is to reduce deformation-induced martensite to austenite under specific conditions and that reverse transformation, revertive recrystallization or discontinuous recrystallization of deformation-induced martensite may occur simultaneously or sequentially [26]. In dynamics, on the one hand, the more deformation-induced martensite is generated, the more prone to reverse transformation in the early stage of annealing, because the more severe deformation-induced martensite will provide more nucleation sites for the austenite grains, therefore, the larger the deformation, the faster the martensite disappears [27]. For example, the volume fractions of martensite before and after annealing of the 90% cold rolled sample changes greatly, because the 90% cold rolled sample has more dislocation cellular martensite, which creates favorable conditions for the formation of nano/ultrafine grain structure in the later stage. On the other hand, with the increase of deformation degree, the newly generated austenite is more refined after annealing, and the generation of austenitic coarse grain is suppressed, thus the larger the cold-rolling deformation is, the more homogeneous the distribution of grain size after annealing is. On the other hand, the inverse phase transformation from α → γ is also driven by the diffusion mechanism [2]. At low annealing temperatures and short annealing times, the nucleation process of austenite is inhibited due to the activation energy barrier. However, when the annealing temperature is increased or the annealing time is prolonged, the nucleation and growth processes of austenite will be effectively promoted. Increasing the annealing temperature provides more thermal energy, which helps to overcome the activation energy barrier during the nucleation process, while prolonging the annealing time increases the effect of diffusion-driven kinetics, allowing the recovery process to proceed more fully.

The microstructural evolution during the annealing process is divided into the following stages: (a) martensitic transformation induced by strain begins to reverse; (b) the reverse-transformed martensite gradually recovers as lath or bulk austenite; (c) new grains are produced to replace the lath or bulk austenite; and (d) the new grains aggregate to form ultrafine grains.

### 4.3. Annealed Mechanical Properties

As can be found from Table 3, when the annealing temperature is in the range of 800–900 °C, the yield strength and tensile strength of the sample decrease rapidly, and the elongation increases rapidly. Afterwards, with the further increase of annealing temperature, the strength value changes little, and the elongation continues to rise. In the previous introduction, the plasticity of 70% cold rolling deformation is very poor, while the plastic of the sample is significantly recovered after high-temperature annealing [28]. This is because the sample, after a large amount of deformation from rolling, has a high density of dislocations and twins and the interactions between them, so that the grains within the larger internal stresses, and after annealing, the grains of the sample begin to recover gradually. When the annealing temperature reaches a certain level, recrystallize grains begin to form inside the sample, which reduces the strength of the sample. After a series of annealing experiments, the results show that the experimental steel has the best property at 850 °C for 30 s, so this temperature is selected as the ideal temperature before the next rolling experiment.

**Figure 11:**
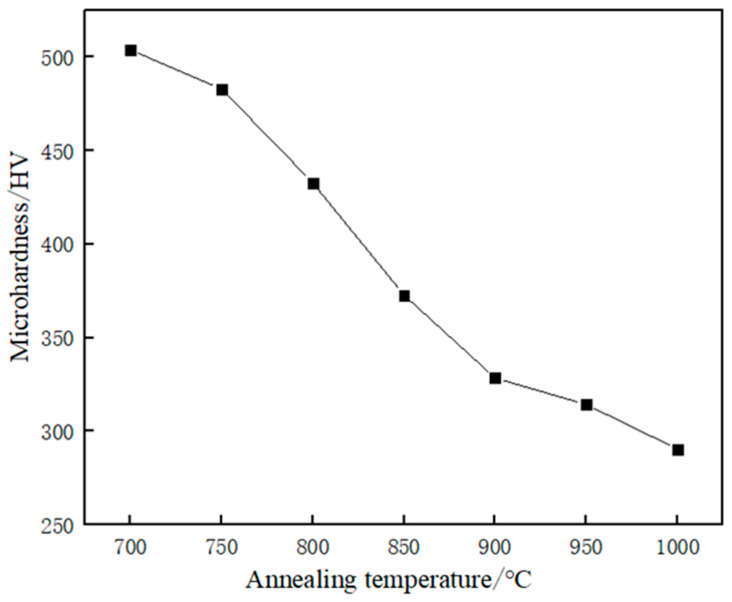
Microhardness of austenitic stainless steel after annealing at different temperature.

Figure 11 shows the microhardness values of austenitic stainless steel after holding for the same time at different annealing temperatures. When the annealing temperature of 700 °C, the sample hardness value of about 503 HV; when the annealing temperature continues to increase to 900 °C, the hardness value of about 328 HV, when the annealing temperature is further increased to 1000 °C, the sample hardness value has been reduced to 290 HV, and with the annealing temperature of 700 °C compared to the reduction of up to about 42%. It can be seen that at the annealing time of 30 s, the microhardness value is in a decreasing trend with the increase of annealing temperature. This is due to the fact that after annealing, the movement between dislocations causes the deformed organization to gradually begin to recover. When the annealing temperature is gradually increased, the subcrystalline structure is formed with the movement and interaction of dislocations. The grains of subcrystalline structure undergo further development and gradually form recrystallised grains; When the annealing temperature continues to rise, it has exceeded the recrystallisation temperature and eventually forms an equiaxed austenitic organization with clear grain boundaries. Dislocations, dislocation cells, etc. disappear, resulting in a decrease in hardness.

After annealing to reduce the strength of the plate, continue to carry out another rolling experiment. After rolling, the thickness of the plate can reach 0.15 mm or so, achieving a yield strength of 1321 MPa. This can meet the strength requirements of the product production, but at this time, the permeability is higher than the application requirements. This weak magnetism will affect the performance of the product, therefore, it is necessary to reduce the permeability by certain control means without affecting its strength requirements. According to the experience and theory of the previous annealing experiments, after rolling again, annealing experiments can be obtained. According to the experience and theory of the previous annealing experiment, the re-annealing experiment after rolling was carried out. Annealing at 870 °C for 20 s can reduce the strength and meet the permeability requirements. At this time, the yield strength is 1238 MPa, the permeability is below 1.02, and the thickness is about 0.15 mm, which meets the production requirements of the metal mask plate.

## 5. Conclusions

By studying the effect of microstructure and mechanical properties of stainless steel after cold rolling and annealing experiments. The following conclusions were drawn:

1. The 305 austenitic stainless steel used in this work has good comprehensive mechanical properties after cold rolling deformation. When the deformation amount is 70%, the yield strength can reach 1058 MPa and the tensile strength can reach 1273 MPa. After annealing, further rolling can make its strength reach 1321 MPa, which meets the strength requirements of production products.
2. In the study of annealing experiment, it is found that the strength and plasticity of the experimental steel are the best after annealing at 850 °C for 30 s, thus this temperature is the most suitable temperature for annealing experiment.
3. The experimental steel after annealing and rolling is annealed again. It can be seen from the experiment that annealing at 870 °C for 20 s can reduce its strength and meet the requirements of permeability. At this time, the yield strength is 1238 MPa and the permeability is below 1.02, which meets the production requirements of metal mask plate.

## Figures and Tables

**Table 1:** Chemical composition of the experimental steel (mass, %).

Elemental	C	Si	Mn	P	S	Cr	Ni	N	Fe
Mass fraction	0.07	0.51	1.05	0.032	0.003	16.15	6.1	0.06	Bal

**Table 2:** Martensite volume fraction l (volume fraction, %).

Deformation	10	30	50	70	90
Martensite volume fraction	15.14	18.06	24.30	31.09	45.17

**Table 3:** Mechanical properties under different annealing conditions.

Annealing Temperature, Time		Yield Strength (MPa)	Tensile Strength (MPa)	Elongation (%)
800 °C	10 s	1012	1203	8.37
800 °C	30 s	960	1132	15.76
800 °C	60 s	903	1050	21.32
850 °C	10 s	897	981	19.14
850 °C	30 s	852	932	43.98
850 °C	60 s	786	817	45.29
900 °C	10 s	702	733	23.50
900 °C	30 s	643	678	52.18
900 °C	60 s	598	612	57.23

## Data Availability

Data can be made available upon reasonable request.
